# Mild orotic aciduria in *UMPS* heterozygotes: a metabolic finding without clinical consequences

**DOI:** 10.1007/s10545-017-0015-9

**Published:** 2017-02-15

**Authors:** Saskia B. Wortmann, Margaret A. Chen, Roberto Colombo, Alessandro Pontoglio, Bader Alhaddad, Lorenzo D. Botto, Tatiana Yuzyuk, Curtis R. Coughlin, Maria Descartes, Stephanie Grűnewald, Bruno Maranda, Philippa B. Mills, James Pitt, Catherine Potente, Richard Rodenburg, Leo A. J. Kluijtmans, Srirangan Sampath, Emil F. Pai, Ron A. Wevers, George E. Tiller, and additional individual contributors, Saskia B. Wortmann, Ron A. Wevers, George E. Tiller, Margaret A. Chen, Roberto Colombo, Alessandro Pontoglio, Bader Alhaddad, Lorenzo D. Botto, Tatiana Yuzyuk, Curtis R. Coughlin, Maria Descartes, Stephanie Grűnewald, McKenna N. M. Kyriss, Bruno Maranda, Philippa B. Mills, James Pitt, Catherine Potente, Emma S. Reid, Richard Rodenburg, Leo A. J. Kluijtmans, Srirangan Sampath, Janet A. Thomas, Paula J. Waters, Susan M. White, Emil F. Pai

**Affiliations:** 1 Department of Pediatrics, Salzburger Landeskliniken (SALK) and Paracelsus Medical University (PMU), Mullner Hauptstrasse 48, 5020 Salzburg, Austria; 2 Institute of Human Genetics, Helmholtz Zentrum Munich, Neuherberg, Germany; 3 Institute of Human Genetics, Technical University Munich, Munich, Germany; 4 Prevention Genetics, Marshfield, WI USA; 5 Institute of Clinical Biochemistry, Faculty of Medicine, Catholic University of the Sacred Heart, Rome, Italy; 6 Center for the Study of Rare Hereditary Diseases, Niguarda Ca’ Granda Metropolitan Hospital, Milan, Italy; 7 Department of Genetics, University of Utah School of Medicine, Salt Lake City, UT USA; 8 Department of Pathology, University of Utah, Salt Lake City, UT USA; 9 ARUP Laboratories, Salt Lake City, UT USA; 10 Department of Pediatrics, University of Colorado Denver, Anschutz Medical Campus, Aurora, CO USA; 11 Departments of Genetics and Pediatrics, University of Alabama School of Medicine, Birmingham, AL USA; 12 Metabolic Medicine Department, Great Ormond Street Hospital for Children NHS Foundation Trust, and UCL Institute of Child Health, London, UK; 13 CHUS Genetic Services, University of Sherbrooke, Sherbrooke, QC Canada; 14 Genetics and Genomic Medicine Programme, UCL Great Ormond Street Institute of Child Health, London, UK; 15 Victorian Clinical Genetics Services, Murdoch Childrens Research Institute, Royal Children’s Hospital, Parkville, Australia; 16 Department of Paediatrics, University of Melbourne, Parkville, Australia; 17 Ambry Genetics, Aliso Viejo, CA USA; 18 Translational Metabolic Laboratory, Department of Laboratory Medicine, Radboud University Medical Center, Nijmegen, The Netherlands; 19 Princess Margaret Cancer Centre, and Departments of Biochemistry, Medical Biophysics, and Molecular Genetics, University of Toronto, Toronto, ON Canada; 20 Department of Genetics, Kaiser Permanente, Los Angeles, CA USA

## Abstract

**Background:**

Elevated urinary excretion of orotic acid is associated with treatable disorders of the urea cycle and pyrimidine metabolism. Establishing the correct and timely diagnosis in a patient with orotic aciduria is key to effective treatment. Uridine monophosphate synthase is involved in de novo pyrimidine synthesis. Uridine monophosphate synthase deficiency (or hereditary orotic aciduria), due to biallelic mutations in *UMPS*, is a rare condition presenting with megaloblastic anemia in the first months of life. If not treated with the pyrimidine precursor uridine, neutropenia, failure to thrive, growth retardation, developmental delay, and intellectual disability may ensue.

**Methods and results:**

We identified mild and isolated orotic aciduria in 11 unrelated individuals with diverse clinical signs and symptoms, the most common denominator being intellectual disability/developmental delay. Of note, none had blood count abnormalities, relevant hyperammonemia or altered plasma amino acid profile. All individuals were found to have heterozygous alterations in *UMPS*. Four of these variants were predicted to be null alleles with complete loss of function. The remaining variants were missense changes and predicted to be damaging to the normal encoded protein. Interestingly, family screening revealed heterozygous *UMPS* variants in combination with mild orotic aciduria in 19 clinically asymptomatic family members.

**Conclusions:**

We therefore conclude that heterozygous *UMPS-*mutations can lead to mild and isolated orotic aciduria without clinical consequence. Partial UMPS-deficiency should be included in the differential diagnosis of mild orotic aciduria. The discovery of heterozygotes manifesting clinical symptoms such as hypotonia and developmental delay are likely due to ascertainment bias.

## Introduction

Establishing the correct and timely diagnosis in a patient with elevated urinary excretion of orotic acid is key to effective treatment. The differential diagnosis of orotic aciduria (OA) is broad, and includes several treatable disorders, like urea cycle defects (especially ornithine transcarbamylase (OTC) deficiency) and uridine monophosphate synthase (UMPS) deficiency (Lichter-Konecki et al 2016). Additionally, OA can be seen in mitochondrial disorders, lysinuric protein intolerance, liver disease, and has been reported in Rett syndrome, malignancies, as a side effect of certain medications and in trauma victims (Jeevanandam et al 1991).

UMPS-deficiency (or hereditary OA, MIM #258900) is a rare inborn error of metabolism (reviewed in Balasubramaniam et al 2014). UMPS (EC 4.1.1.23), encoded by the *UMPS* gene, is a bifunctional protein in the de novo pyrimidine synthesis. In the first reaction, orotate phosphoribosyltransferase (OPRTase) converts orotate to orotidine monophosphate. In the second step, orotidine decarboxylase (OMPdecase) decarboxylates orotidine monophosphate to uridine monophosphate. Patients with UMPS-deficiency reported to date presented with megaloblastic anemia in the first months of life. If untreated this disorder can lead to neutropenia, failure to thrive, growth retardation, sparse hair and nail growth, developmental delay, intellectual disability, and epilepsy. Gross excretion of orotic acid may also cause crystalluria in later life (Bailey 2009; Grohmann et al 2015).

The *UMPS* gene consists of six exons, and encodes a protein that is 480 amino acids in length (Suchi et al 1997). In mammals and yeast, the active enzyme is a dimer (Wittmann et al 2008). The first N-terminal 214 amino acids comprise OPRTase, while the C-terminal 258 amino acids comprise OMPdecase (Suttle et al 1988). Biallelic missense mutations which lead to amino acid substitutions are a presumed common cause of disease; biochemical studies in cell lines from UMPS-deficient patients have demonstrated decreased steady-state levels of the enzyme, impaired substrate binding, and reduced catalytic efficiency (Winkler and Suttle 1988; Perry and Jones 1989; Suchi et al 1997).

Here we discuss the clinical relevance of heterozygosity for *UMPS* variants and mild OA by presenting a case series of 11 index cases and 18 healthy family members.

## Individuals and methods

**Metabolic screening** included complete blood count, blood gas analysis, serum glucose, electrolytes and lactate, plasma ammonia and amino acids, urinary organic acids, purines and pyrimidines and was (repetitively) performed using standard methods.

**Enzyme activity in erythrocytes** OPRTase and OMPdecase activities were determined in lysates of fresh erythrocytes as previously described (Krungkrai et al 2001)

### Genetic investigations

*Exome sequencing* of the individuals III-6, III-7, IV-5, and IV-6 from pedigree 2, I8, I10, and I11 was performed as previously described (Wortmann et al 2015). *Bidirectional Sanger sequencing* of all six *UMPS* (NM_000373.3 (hg19)) coding exons plus ∼20 bp of flanking non-coding intronic DNA was performed in individuals I2-I11 and family members on genomic DNA using standard methods (primer sequences and PCR conditions are available upon request). *SNP array* analysis (Affymetrix SNP 6.0) of individuals I1, I4, and I8 as well as *karyotyping* of I4 was performed by standard methods. Whole genome copy number screening was also performed for individual I8 (CytoSure ISCA v2 4x180k array, Oxford Gene Technology). *Gene-specific deletion/duplication testing of UMPS* of individuals I4, I6, and I7 was performed via high density gene-centric array CGH (Prevention Genetics, details available upon request). *Gene panel sequencing (GPS)* of individual I12 was performed using a targeted panel of genes causing inborn errors of metabolism (details available upon request).

### Molecular modeling of UMPS variants

The models presented are based on PDB-entries 2WNS and 3BGG (www.rcsb.org/pdb/) for the OPRTase and OMPdecase domains of human UMPS, respectively. Figure 2 was prepared using PyMOL 1.6.0.0 (The PyMOL Molecular Graphics System, Version 1.8 Schrödinger, LLC).

## Clinical cohort

The 11 index individuals (I1….I13) were referred for various signs and symptoms as detailed below; all were shown to have OA (summarized in Table 1). Additionally, one pedigree (Ped2) is described in which the index case did not have OA, but several family members. Of note, none of the investigated index patients nor their family members had relevant anemia or neutropenia, hyperammonemia, growth delay or failure to thrive.

**Table 1:** Clinical and metabolic findings and *UMPS* carrier status of all individuals

	*UMPS* mutation	Clinical signs and symptoms	Urinary orotic acid (umol/mmol creatinine)	Max. factor of urinary orotic acid elevation	Orotate/ orotidine ratio
I1	+	Neonatal encephalitis, feeding difficulties, mild LD	30, 31 (RR<4.9)	6.3	n/a
M1, F1	–	NRMC	n/a		n/a
Ped2	+	12 x NRMC	22–43.1 (RR 0.05–3)	14.3	n/a
Ped2	–	13 x NRMC, 1x ID/DD, infantile seizures	<0.2–3 (RR 0.05–3)		
I3	+	ID	10.5 (RR <1.5 +/−0.4)	5.3	n/a
M3	+	n/a	38 (RR 4.9 +/−1.8)	5.7	n/a
I4	+	DD, myoclonic seizures	17.3 (RR 1–3.2)	5.4	13.5
I5	+	Mild LD	15.5–49 (RR <1.5)	6 (after allopurinol 32.3)	5–15.2
M5	+	NRMC	7.3 (RR <1.5)	5	n/a
F5	–	NRMC	Unmeasurably low		n/a
I6	+	FTT, speech delay	34–53 (RR <4.9)	10.8	6.4
M6	+	mild ID	13.8 (RR <4.9)	2.8	n/a
S6	–	NRMC	<4.9 (RR <4.9)		
I7	+	DD, dysmorphic features, hypotonia	40 (RR <8)	5	3.36
F7	–	NRMC	n/a		n/a
M7	+	NRMC	n/a		n/a
S7	+	NRMC	7.8 (RR <3.4)	2.32	n/a
I8	+	DD, joint hypermobility	7–16, after allopurinol 44–182 (RR <4)	4 (after allopurinol 45)	1.6–7.3
M8	+	NRMC	4 (RR <4)	–	5
I9	+	DD	18–35 (RR <7)	5	n/a
I10	+	DD, hypotonia, joint hypermobility, exercise intolerance	14–23 (RR <4)	5.75	5.4–8.5
M10	+	NRMC	19 (RR <4)	4.75	n/a
F10	–	NRMC	<4 (RR <4)	–	n/a
I11	+	diabetes mellitus I, autism, Mauriac syndrome	11 (RR <4)	2.75	8.5
M11	–	NRMC	<2 (RR <4)	–	n/a
F11	+	NRMC	10 (RR <4)	2.5	3.4
I12	+	NRMC	9, 11, 11, 24, 16, 28, 42, 48, 39, 40, 44, 34 (RR <5)	9.6	n/a
M12	n/a	NRMC	<5 (RR <5)		n/a
F12	n/a	NRMC	<5 (RR <5)		n/a

*DD* developmental delay, *F* father of, *FTT* failure to thrive, *I* individual, *ID* intellectual disability, *LD* learning disability, *M* mother of, *n/a* not available, *NRMC* no relevant medical complaints, *Ped* pedigree, *RR* reference range, *S* sibling of

**Fig. 1:**
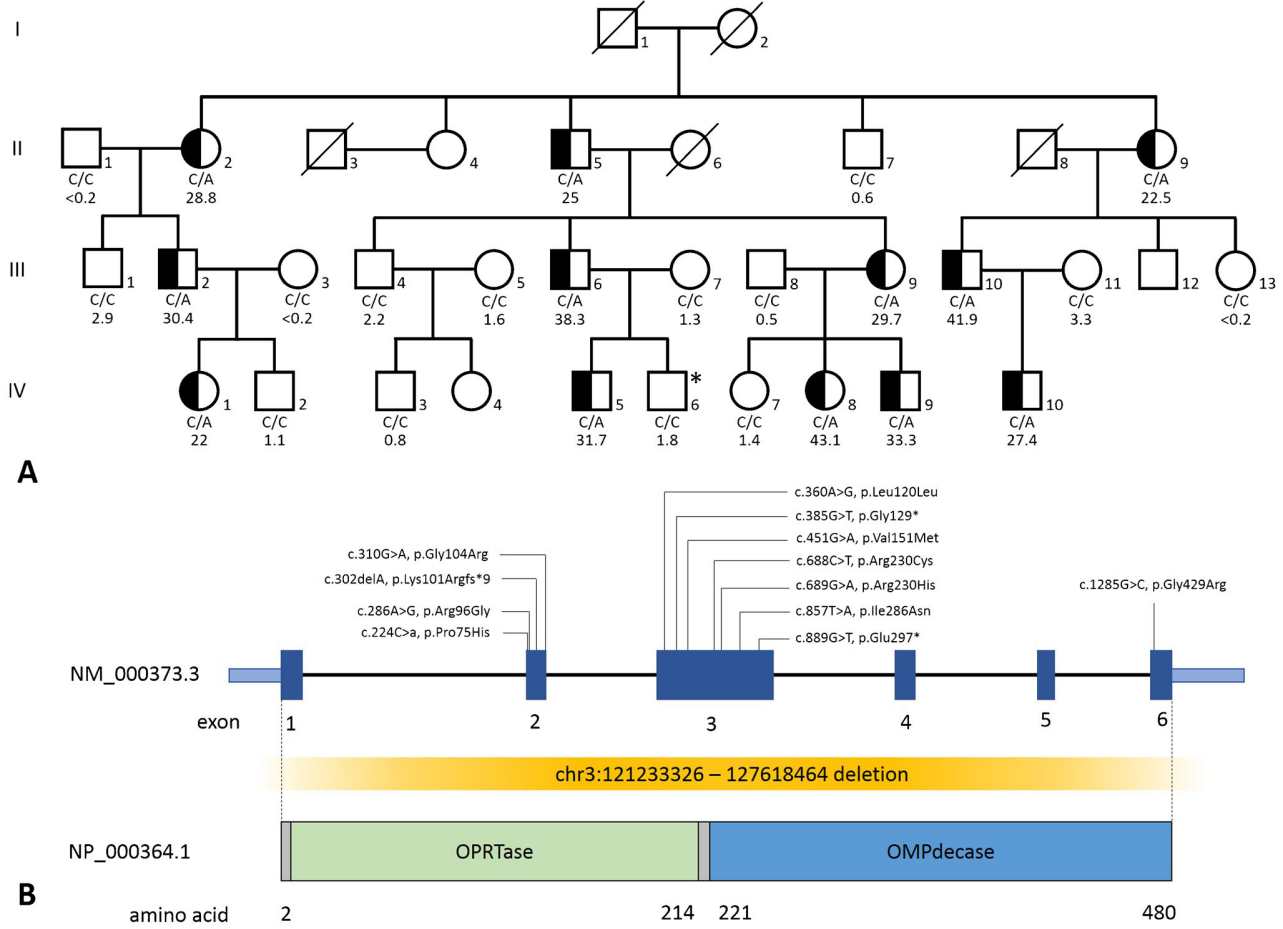
Pedigree of family P2 and structure of the human *UMPS* gene including variants described in all individuals. **a** Half-filled symbols indicate ascertained orotic aciduria heterozygous subjects. An *asterisk* (*) denotes the “index” case. Carrier status for UMPS p.Pro75His mutation (genotype C/A at *UMPS* c.224 locus) is reported under the symbols, followed by the level of urinary orotic acid (μmol/mmol creatinine; reference range: 0.05–3). **b** Structure of the human *UMPS* gene including variants described in all individuals. *OMPdecase* orotidine decarboxylase, *OPRTase* orotate phosphoribosyltransferase

*in pedigree 2 26 family members were tested (see Fig. 1 for individual values)

**Individual I1**, an Australian female born at term, presented neonatally with a clinical picture of encephalitis. Metabolic screening showed mild transient hyperammonemia and persistent OA without any other biochemical abnormalities. Apneic episodes resolved over 5 days and she was discharged on day 9. Brain MRI showed isolated partial agenesis of the corpus callosum. She had feeding difficulties as an infant and dysplastic hips. Repetitive metabolic screening showed no abnormalities other than consistent isolated OA. Currently, at the age of 6 years, she has some learning difficulties and is educated in a mainstream school with additional support. Both parents (M1, F1) had no relevant medical complaints (NRMC); urine of parents was not tested. **Pedigree P2** (Fig. 1a)**:** The parents of this “index” male (IV:6) with intellectual disability and infantile seizures were seeking genetic advice. They agreed to participate in a study for establishing reference values for urinary purines and pyrimidines measurements. This lead to detection of OA in the Italian father (III:6) and the older brother (IV:5) of this “index” who both had NRMC. The “index” himself and his Croatian mother (III:7) did not show OA. OPRTase activities were within the normal range for all four family members (father 0.037 ± 0.008, mother 0.312 ± 0.087, older son 0.265 ± 0.079, “index” 0.059 ± 0.008; reference range 0.288 ± 0.073 nmol/mg protein/h). OMPdecase activities were in the heterozygous range in the father (0.073 ± 0.011) and older son (0.096 ± 0.008), and normal in the mother (0.395 ± 0.091) and the “index” (0.424 ± 0.098; reference range 0.409 ± 0.094 nmol/mg protein/h). Finally, a total of 12 family members were shown to have OA and another 14 did not have OA. Beside the “index” (without OA), NRMC were reported for the other family members. **Individual I3** (data extracted from Suchi et al 1997; Imaeda et al 1998), a Japanese male of unreported age, was evaluated for intellectual disability. He and his healthy mother (M3) were both reported to have OA. In both individuals, the OPRTase and OMPdecase enzymes measured in red blood cells were reported to be reduced but in the heterozygous range (details not given). No details of the father were provided. **Individual I4** presented at the age of 7 months with developmental delay. At the age of 16 months, this Caucasian male had his first myoclonic seizures; a brain MRI at the same age was unremarkable. Mild OA was noted, but further metabolic screening was unremarkable. Both parents (M4, F4) had NRMC. **Individual I5**, a 6 year old Hispanic male, presented as a premature infant (30 weeks of gestation) with transient hyperammonemia. Urine organic acid profile revealed persistent mild OA. Development has been normal, except for a mild learning disability (WISC-V: average global IQ, lower score for working memory and risc score for inattention/impulsivity). His mother (M5) also showed mild OA, but his father (F5) did not. Both parents had NRMC. **Individual I6**, a 20 month old Caucasian male, presented with failure to thrive, multiple food allergies, and speech delay. Metabolic screening was unremarkable except for persistent OA. The mother (M6) has mild cognitive impairment and a moderate degree of OA; a healthy male maternal half-sibling (S6) did not exhibit OA. The father was not available for evaluation. **Individual I7**, a 10 month old Caucasian male, presented with dysmorphic features, hypotonia, developmental delay, and macrocephaly. Past medical history was notable for resuscitation at birth after an unremarkable pregnancy. His history further included strabismus, dysphagia, gastroesophageal reflux, and recurrent abdominal pain, as well as left talipes equinovarus. Urinary organic acid analysis revealed repeatedly mild OA, once with concomitant slightly elevations of glycolic acid, oxalic acid and uracil. Urinary purine and pyrimidine analysis was initially normal, however, repeated testing showed an elevated orotic acid. At 3 years of age the patient was healthy and making developmental gains. The patient’s healthy brother (S7) also showed mild OA. Neither parent (M7, F7) had any relevant medical complaints. **Individual I8**, a 1 year old Dutch female, presented with global developmental delay, generalized joint hypermobility and persistent OA. Over time, her development showed a satisfactory catch-up development, and at 5 years of age she is attending regular school. She still has joint hypermobility. The mother (M8) had NRMC and no OA. The biological father was an anonymous sperm donor. **Individual I9** 2.5 year old French-Canadian female, presented with global developmental delay. Her past medical history was otherwise unremarkable. The metabolic screening was consistently normal with exception of OA. The family history was unremarkable and parents (M9, F9) had NRMC. **Individual I10**, a 1 year old Dutch male, presented with generalized muscular hypotonia, joint hypermobility, and weakness of the ocular, oral, and limb girdle musculature. A muscle biopsy showed fiber type disproportion. Metabolic screening revealed isolated OA with no other abnormalities. At last evaluation (age 7 years), he showed an increase in muscle strength and improved motor development. The parents are healthy, the mother (M10) exhibited mild OA; urine analysis of the father (F10) was unremarkable. **Individual I11**, a 16 year old Dutch male with autism and poorly controlled type I diabetes mellitus, presented with liver failure. Elevated transaminases and lactate levels were evident on admission. The diagnosis of Mauriac syndrome (secondary hepatic glycogenosis in poorly controlled diabetes mellitus type I) was made and he fully recovered with improved diabetes control. Metabolic screening showed isolated OA. Both parents are healthy, the father (F11) exhibited mild OA; urine analysis of the mother (M11) was unremarkable.

**Individual I12**, the second child of healthy unrelated Polish parents, was brought to attention at birth. His older brother was born prematurely and died neonatally due to hyperammonaemia of unknown cause. This sibling was treated prospectively from birth with a low protein diet, sodium benzoate, and arginine supplements. There were no clinical concerns regarding this child, however urinary organic acid analysis showed persistent mild OA. Around 6 months of age his diet was liberalized and medication was ceased. At last evaluation (age 3 years) he was neuro-developmentally normal. Both parents (M12, F 12) have normal urine organic acids and NRMC.

## Results

### Genetic results (Table 2, Figs. 1 and 2)

**Table 2:** Genetic findings of all individuals with *UMPS* variants

	Alteration in *UMPS* c.DNA	Predicted amino acid alteration	Predicted effect	Allele frequency in EXAC browser	PolyPhen2	SIFT	Mutation taster	Conservation
I1	6.4 Mb deletion	*UMPS* deleted	no protein					
Ped2	c.224C>A	p.Pro75His	missense	Not listed	deleterious	deleterious		
I3, M3	c.286A>G	p.Arg96Gly	missense	0.00004318	Benign	not tolerated	disease causing	D. melanogaster
I4	c.302delA	p.Lys101Argfs*9	frameshift and premature stop; possible nmd	not listed				
I5, M5	c.310G>A	p.Gly104Arg	missense, alternative splicing of the exon	not listed	probably damaging	not tolerated	disease causing	“well”
I6, M6	c.360A>G	p.Leu120Leu	synonymous, possible weak cryptic splice donor site	not listed				
I7, M7, S7	c.385G>T	p.Gly129*	premature stop; possible nmd	0.000008237				
I8, M8	c.688C>T	p.Arg230Cys	missense	not listed	probably damaging	not tolerated	disease causing	C. elegans
I6, M6	c.689G>A	p.Arg230His	missense	not listed	probably damaging	not tolerated	disease causing	D. melanogaster
I9	c.857T>A	p.Ile286Asn	missense	0.00004131	probably damaging	not tolerated	disease causing	D. melanogaster
I10, M10, I11, F11	c.889G>T	p.Glu297*	premature stop; possible NMD	not listed				
I3, M3	c.1285G>C	p.Gly429Arg	missense	0.00005766	probably damaging	not tolerated	disease causing	C. elegans
I12	c.451G>A	p.Val151Met	missense	0.000008238	probably damaging	not tolerated	disease causing	D. melanogaster

*I* individual, *NMD* nonsense mediated RNA decay, *M* mother of, *Ped* pedigree, *S* sibling of

**Fig. 2:**
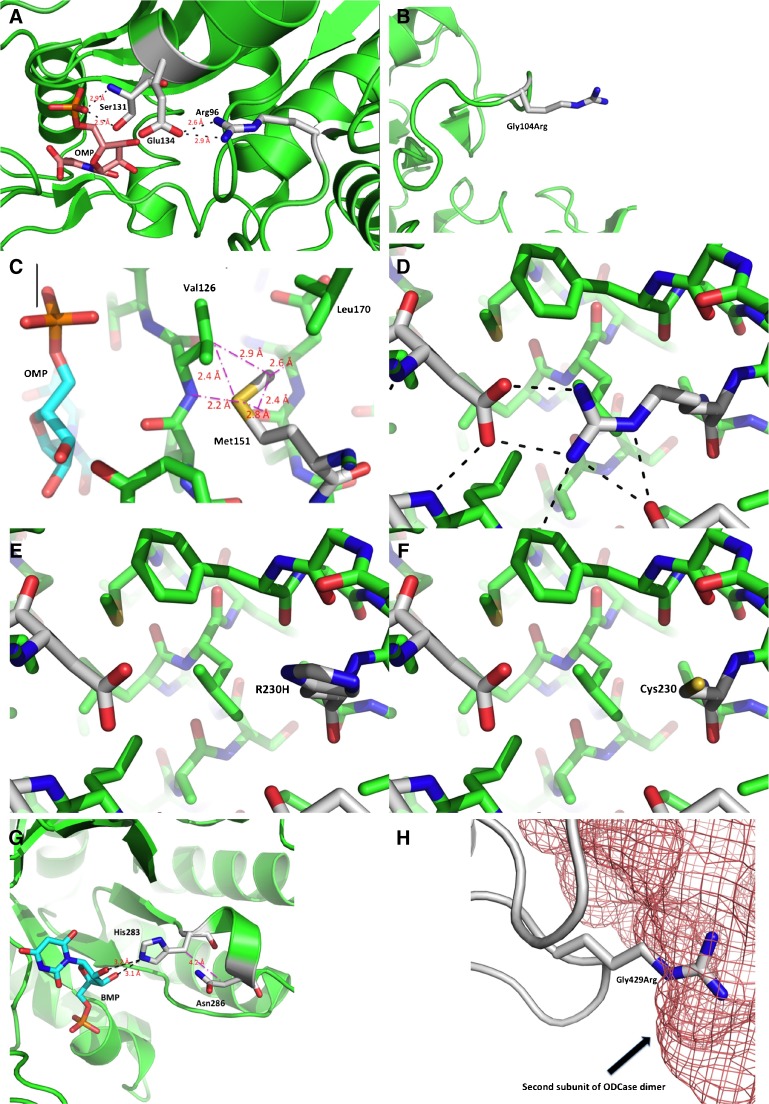
Molecular modeling of UMPS missense variants. **a Arg96Gly:** Arg96 binds to Glu134, which is part of a short helix and in close proximity to Ser131 that binds to the OMP 5′-phosphate (OMP) substrate. The same serine residue also interacts tightly with Lys29, which in turn binds to OMP. Mutation of Arg96 to Gly destroys the interaction with Glu134, with potential effects on helix placement and substrate-enzyme contacts. **b Gly104Arg:** Gly104 is part of a ß-turn located at the surface of the protein molecule. The side chain of the substituted arginine would point into the solvent with no obvious major direct structural effects. However, it is possible that the ß-turn could become destabilized, with potential consequences for folding and stability of the protein molecule. **c Val151Met:** Replacing the short side chain of Val151 with a bulky methionine would lead to a large number of contacts shorter than the sum of van der Waals radii between residues of the hydrophobic cluster surrounding this residue with obvious effects on the stability of the protein fold and side chain orientation. One member of the hydrophobic cluster is Val126, which is right next to the substrate OMP; any shift away from valine’s present position would interfere with substrate binding. **d Arg230:** Arg230 is on the surface of the protein molecule, but its side chain points into the body of the protein and participates in a large number of electrostatic and hydrogen-bonding interactions. **e Arg230His:** Substituting Arg230 (an important anchor point for several secondary structure elements) with His would lead to a loss of all these interactions. **f Arg230Cys:** As indicated in Fig. 2e for the Arg230His mutation, replacing the long charged side chain of Arg230 (see Fig. 2d) with the much smaller Cys leads to the same loss of interactions. **g Ile286Asn:** Introducing an asparagine residue at this position places a polar side chain close enough to neighboring His283 to make hydrogen bonding a possibility. The subsequent rotation of the His283 ring could destroy binding of His283 to the ribose moiety in OMP. **h Gly429Arg:** Gly429 is located on the dimer interface of the protein, as well as part of a loop surrounding the orotidine moiety of OMP (Wittmann et al., 2008). The substitution of arginine at this residue could interfere with dimerization, and possibly impact substrate binding by Gln241

SNP array of **I1** showed a de novo 6.4 Mb deletion on chromosome 3 (chr3:121233326–127618464) containing more than 40 RefSeq genes, including *UMPS*, making it likely to be contributing to the patient’s phenotype. It did not contain any genes known to cause agenesis of the corpus callosum when deleted. In four individuals (I4, I7, I10, I11) and four healthy family members (M7, S7, M10, F11), three different heterozygous alterations *UMPS* were found which were predicted to lead to a premature stop and thereby loss of normal protein function via protein truncation or nonsense-mediated mRNA decay (NMD). In I4 c.302delA (p.Lys101Argfs*9) was found; karyotyping, SNP array, and gene-specific deletion/duplication testing of *UMPS* via high-density gene-centric array CGH did not reveal abnormalities. No family members were tested. I7 harbored the c.385G>T (p.Gly129*) variant in *UMPS*, which was maternally inherited and also found in the healthy brother (S7). In both I10 and I11 (coming from the same region of the Netherlands), a c.889G>T (p.Glu297*) variant was found (maternally inherited in I10; paternally in I11). In six individuals (I3, I5, I6, I8, I9, I12) and 16 healthy family members (M3, M5, M6, M7, *n* = 12 from pedigree 2) seven different heterozygous missense alterations were detected. In pedigree 2, 12 family members (Fig. 1) tested positive for the c.224C>A (p.Pro75His) variant. In five cases the variant was derived maternally, in four cases paternally and in three cases it is unknown. In I3 and his healthy mother M3 (7,13) two variants c.286A>G (p.Arg96Gly) and c.1285G>C (p.Gly429Arg) in *UMPS* were found, therefore likely to be on the same allele. In I5 the variant c.310G>A (Gly104Arg) was detected, I6 harbored c.689G>A (p.Arg230His), in I7 c.688C>T (p.Arg230Cys) was found, both were maternally inherited. In I9 c.857T>A (p.Ile286Asn) and in I11 c.451G>A (p.Val151Met) were detected; both parents were not tested. The missense alterations found were predicted to be pathogenic by different software programs (PolyPhen-2, SIFT, MutationTaster). All amino acids affected were well-conserved from H. sapiens (down) to D. melanogaster or to C. elegans. I6 was also heterozygous for a second maternally inherited undocumented variant, c.360A>G (p.Leu120Leu), a synonymous change. In individuals 4, 6, and 7, additionally copy number variations in *UMPS* were excluded. Most of the variants were not listed in the Exac browser; for those listed, the allele frequencies were below 0.001 (Table 2).

#### Molecular modeling of UMPS variants

The effects of missense mutations in the *UMPS* gene on the protein conformation are illustrated in Fig. 2 and described in the legend of the figure.

## Discussion

Establishing the correct and timely diagnosis in a patient with OA is key to effective treatment. The more common urea cycle disorders require a protein-restricted diet, prevention of catabolic situations, and treatment with ammonia-detoxificating medication (Lichter-Konecki et al 2016). The very rare UMPS-deficiency has been treated successfully with uridine compounds (Becroft and Phillips 1965; Alvarado et al 1988; Bensen et al 1991). In addition, the correct diagnosis has a substantial impact on the counseling of the patient and the family, including discussion of recurrence risk.

The literature does not provide us with a reliable estimate of the incidence of OA due to UMPS-deficiency nor the prevalence of *UMPS-*mutation carriers. A single survey (Rogers et al 1975) described the results of screening over 1000 “mentally retarded” patients for organic acidurias. Of nine subjects with OA, two were found to have decreased OPRTase and OMPdecase enzyme activities, which were also documented in a parent of each child. Early reports of children with OA without megaloblastic anemia, abnormal growth, or developmental delay (Tubergen et al 1969) may well represent *UMPS*-mutation carriers, as they were reported in the pre-molecular age.

Due to lack of clinical information, present-day databases do not shed light on the incidence of OA either. There are over 70 entries in the ClinVar/dbVar databases with genomic deletions that include *UMPS*, but only seven are described as “pathogenic.” Those deletions range from 4 to 94 Mb in size, and include from 45 to 707 genes. No clinical or biochemical information is available. There are 48 missense variants listed in Ensembl for which additional information is available, but only the three mutations reported by (Suchi et al 1997) were identified as pathogenic. These three mutations are also the only pathogenic mutations among the 150 missense variants listed in the ExAC browser as well as the 155 missense variants listed in dbSNP. Since many of the variants listed may be benign, it is clear that these databases are failing to support clinicians.

As demonstrated in the 11 individuals described, OA is a prominent and challenging biomarker that can mislead the diagnostic team of metabolic physicians and laboratory staff, potentially delay arrival at the correct diagnosis, increase diagnostic costs, and generate unpleasant and often unnecessary investigations in children (e.g., repetitive ammonia and OA testing, allopurinol loading test in I8, *OTC* gene sequencing etc.). The individuals reported herein all had urinary OA levels (elevated 2.5–14.3 times of upper reference limit) that were relatively constant unlike in OTC deficiency, in which levels vary greatly (Lichter-Konecki et al 2016) and are usually much lower than in classical UMPS-deficiency. Furthermore, the individuals in our cohort had non-informative plasma amino acid profiles and no other abnormal laboratory findings, especially no relevant hyperammonemia nor hematological abnormalities. The *UMPS*-mutations found in these individuals occurred in both domains of the enzyme (OPRTase and OMPdecase). Mutations in both parts of the enzyme led to similar mildly increased urinary orotic acid concentrations.

Metabolic screening in the individuals described (Table 1) was pursued most often due to delayed development (*n* = 6), which is a common practice (van Karnebeek et al 2014). Other indications were muscular hypotonia, suspicion of a mitochondrial disorder, neonatal encephalitis, liver failure, and unexplained neonatal death of a sibling with hyperammonemia. In all cases the clinical signs and symptoms were non-specific and together with the metabolic screening results were not suggestive of a known disorder associated with OA. Additionally, OA was also present in 18 family members harboring the same genetic variants as the probands. Only one carrier parent (M8) did not exhibit OA.

A previous study identified several individuals with benign, persistent OA and heterozygosity for an *UMPS* defect was suggested as one of several possible reasons for these observations (Carpenter et al 1997). Our findings confirm that heterozygous alterations in *UMPS* can indeed lead to mild and isolated OA without clinical consequences.

Balasubramaniam et al (2014) suggested that calculation of the orotate/orotidine ratio may reflect the effect of mutations on the relative activities of ORPTase and OMPdecase. We did not see a correlation in this ratio in our series of patients. Urinary orotidine levels were available in six patients in our cohort. Four of these patients harbored mutations leading to premature termination of translation, which should impact both enzymatic activities. The other two patients harbored different missense mutations in the same codon (230) of UMPS (which lies in the OMPdecase coding region). These are predicted to interfere with electrostatic interactions within the OMPdecase region but not necessarily alter dimerization. There were overlapping orotate/orotidine ratios between these two groups of patients, hence precluding any biochemical correlation.

Therefore, we conclude that heterozygous alterations in *UMPS* can lead to mild and isolated OA without clinical consequences. The prevalent presentation of developmental delay in our patient population may be due to ascertainment bias. Five (I3, I4, I5, I7, I8, I10) of six individuals with some degree of developmental delay or intellectual disability had maternally derived mutations. This may suggest that in utero exposure to elevated levels of orotic acid and/or decreased pools of pyrimidines may impact neurologic development. This theory, however, is not corroborated by the fact that the siblings S6 and S7, as well as five individuals from pedigree 2, with maternally derived mutations do not have any relevant medical complaints.

In summary, heterozygous rare variants in *UMPS* should be considered in all patients with isolated mild OA before pursuing further diagnostic studies. Alternatively, measuring urinary orotic acid levels in parents is a rapid and inexpensive means of determining clinical significance of OA in children.

## Abbreviations

OA: Orotic aciduria
OTC: Ornithine transcarbamylase
UMPS: Uridine monophosphate synthase
OPRTase: Orotate phosphoribosyltransferase
OMPdecase: Orotidine decarboxylase
